# Comparative Analysis of Pediatric COVID-19 Infection in Southeast Asia, South Asia, Japan, and China

**DOI:** 10.4269/ajtmh.21-0299

**Published:** 2021-06-15

**Authors:** Judith Ju Ming Wong, Qalab Abbas, Soo Lin Chuah, Ririe Fachrina Malisie, Kah Min Pon, Tomohiro Katsuta, Hongxing Dang, Pei Chuen Lee, Muralidharan Jayashree, Rehena Sultana, Quratulain Maha, Chin Seng Gan, Naoki Shimizu, Feng Xu, Swee Fong Tang, Luming Shi, Jan Hau Lee, Koh Cheng Thoon, Chee Fu Yung, the PACCOVRA Investigators of the PACCMAN Research Group

**Affiliations:** 1 Children’s Intensive Care Unit, Department of Pediatric Subspecialties, KK Women’s and Children’s Hospital, Singapore;; 2 Duke-NUS Medical School, Singapore;; 3 SingHealth Duke-NUS Global Health Institute, Singapore;; 4 Pediatric Critical Care Medicine, Aga Khan University, Karachi, Pakistan;; 5 Department of Pediatrics, University Malaya Medical Centre, University of Malaya, Kuala Lumpur, Malaysia;; 6 Murni Teguh Memorial Hospital, Medan, Indonesia;; 7 Pediatric Intensive Care Unit, Hospital Pulau Pinang, Pulau Pinang, Malaysia;; 8 Infectious Disease Service, Department of Pediatrics, St. Marianna University School of Medicine, Kanagawa Prefecture, Japan;; 9 Critical Care Treatment Center and Intensive Care Medicine, Children’s Hospital of Chongqing Medical University, Chongqing, China;; 10 Pediatric Intensive Care Unit, Hospital Canselor Tuanku Muhriz, Kuala Lumpur, Malaysia;; 11 Pediatric Intensive Care and Emergency Units, Advanced Pediatrics Centre, Postgraduate Institute of Medical Education and Research, Chandigarh, India;; 12 Center for Quantitative Medicine, Duke-NUS Medical School, Singapore;; 13 Medical College, Aga Khan University, Karachi, Pakistan;; 14 Pediatric Intensive Care Unit, Department of Pediatrics, St. Marianna University School of Medicine, Kanagawa Prefecture, Japan;; 15 Singapore Clinical Research Institute, Consortium for Clinical Research and Innovation, Singapore;; 16 Infectious Disease Service, Department of Pediatrics, KK Women’s and Children’s Hospital, Singapore;; 17 Lee Kong Chian School of Medicine, Imperial College, Nanyang Technological University, Singapore

## Abstract

There is a scarcity of data regarding coronavirus disease 2019 (COVID-19) infection in children from southeast and south Asia. This study aims to identify risk factors for severe COVID-19 disease among children in the region. This is an observational study of children with COVID-19 infection in hospitals contributing data to the Pediatric Acute and Critical Care COVID-19 Registry of Asia. Laboratory-confirmed COVID-19 cases were included in this registry. The primary outcome was severity of COVID-19 infection as defined by the World Health Organization (WHO) (mild, moderate, severe, or critical). Epidemiology, clinical and laboratory features, and outcomes of children with COVID-19 are described. Univariate and multivariable logistic regression models were used to identify risk factors for severe/critical disease. A total of 260 COVID-19 cases from eight hospitals across seven countries (China, Japan, Singapore, Malaysia, Indonesia, India, and Pakistan) were included. The common clinical manifestations were similar across countries: fever (64%), cough (39%), and coryza (23%). Approximately 40% of children were asymptomatic, and overall mortality was 2.3%, with all deaths reported from India and Pakistan. Using the multivariable model, the infant age group, presence of comorbidities, and cough on presentation were associated with severe/critical COVID-19. This epidemiological study of pediatric COVID-19 infection demonstrated similar clinical presentations of COVID-19 in children across Asia. Risk factors for severe disease in children were age younger than 12 months, presence of comorbidities, and cough at presentation. Further studies are needed to determine whether differences in mortality are the result of genetic factors, cultural practices, or environmental exposures.

## INTRODUCTION

The infection rates of novel severe acute respiratory syndrome coronavirus 2 (SARS-CoV-2) are disproportionately low in children compared with adults since its estimated emergence in November 2019. The first pediatric study from the Chinese Center for Disease Control and Prevention (*N* = 2,143) reported a relatively low incidence of severe/critical cases (5.9% among infected cases) and one death.^1^ However, severe or critical illness and death among children with coronavirus disease 2019 (COVID-19) can occur. A dedicated report from 46 North American pediatric intensive care units (ICUs) identified 48 critically ill patients with pediatric COVID-19 infection.^2^ The Italian Integrated COVID-19 Surveillance System identified 3,836 children with COVID-19 infection, of which 86 of 3,836 (2.2%) developed severe/critical disease and four (0.1%) died.^3^ In a meta-analysis of 7,780 pediatric COVID-19 patients, complications such as shock, disseminated coagulopathy, kidney injury, and need for mechanical ventilation occurred in less than 1% and death in 0.1% of children.^4^ Among the 131 studies identified during this meta-analysis, there was a conspicuous absence of data on COVID-19 in children, especially from southeast and south Asia.^4^ There have also been reports from Europe^5,6^ and the United States^7^of a Kawasaki-like syndrome affecting children who have recently recovered from COVID-19. This has been termed multisystem inflammatory syndrome in children (MIS-C), and deaths have been reported from this syndrome. However, to date, apart from India^8^ and Pakistan,^9^ no reports of MIS-C have been reported from southeast Asia as well as China, Japan, and South Korea, which may indicate a possible geographic or genetic variation in risk to COVID-19.

This study aims to describe and compare the clinical epidemiology of pediatric COVID-19 infection in southeast Asia (Singapore, Malaysia, and Indonesia), south Asia (India and Pakistan), Japan, and China. We also identify risk factors for severe COVID-19 among children in the region.

## MATERIALS AND METHODS

We conducted an observational study of children with COVID-19 infection admitted to hospitals contributing data to the Pediatric Acute and Critical Care COVID-19 Registry of Asia (PACCOVRA). This is an ongoing registry (www.clinicaltrial.gov registration no. NCT04395781) within the Pediatric Acute and Critical Care Asian Medicine Network. Characteristics of participating centers have been described elsewhere.^10^ Reporting was conducted in compliance with the Strengthening the Reporting of Observational Studies in Epidemiology guidelines.^11^ Institutional review board approval was obtained from all participating hospitals, and waiver of consent was granted at all sites.

### Patients.

Patients were identified through administrative databases or hospital admission logs of suspected/confirmed COVID-19 cases. A subset of the cases (*n* = 70) from Singapore^12^ and India were published previously.^13^ All infants and children from birth to 21 years at the time of diagnosis were included. A confirmed COVID-19 case was defined as a patient with laboratory confirmation of SARS-CoV-2 infection. Laboratory confirmation was based on any one of the following: 1) nasopharyngeal (or endotracheal if intubated) aspirate for COVID-19 nucleic acid reverse transcriptase–polymerase chain reaction (RT-PCR), 2) a positive serum-specific COVID-19 IgM test, or 3) demonstration of seroconversion of serum-specific COVID-19 IgG (negative to positive) or a 4-fold rise in IgG titers.

### Data extraction.

A centralized, online COVID-19 standardized database was set up using the Research Electronic Data Capture system, with the main coordinating center in Singapore, and was accessible to all site investigators.^14^ Data were extracted from medical records and entered by trained staff at each participating site. Data extracted included epidemiological, clinical, laboratory, and outcome data. Baseline data were captured on presentation to the hospital. Outcome data were captured on discharge from the hospital and refer to the highest severity of illness or level of support throughout the disease course. The centralized registry database was queried on November 26, 2020.

### Outcomes.

The primary outcome was severity of COVID-19 infection as defined by the WHO: mild, moderate, severe, or critical.^15^ Secondary outcomes included the need for any respiratory support (including oxygen), organ dysfunction (defined by the International Pediatric Sepsis Consensus Conference),^16^ and hospital mortality.

### Statistical analysis.

Patients who fulfilled the WHO definition for confirmed COVID-19 infection were treated as binary data and classified into two broad severity categories: mild/moderate and severe/critical. All the variables were summarized in terms of the previously mentioned severity categories. Categorical and continuous variables were presented as counts (percentages) and median (interquartile range), respectively. Associations between severity groups and other categorical variables were evaluated using the χ^2^ test, whereas the association between continuous variables were tested using the Kruskal-Wallis test. In addition to laboratory investigations, which were not routine at all sites, missing data were minimal (< 10%) for all variables. No imputation for missing data was done. Univariate and multivariable logistic regression models were used to identify risk factors for severe/critical disease. To account for correlated patient responses within countries, models were analyzed with PROC GENMOD with binomial distributions and logit link functions. Quantitative associations from GEMOD analysis were expressed as odds ratios with a 95% CI. Modeling was performed using a manual backward elimination method with an exclusion criterion set at *P* > 0.1. Adjustment for country was fixed in the multivariable model. All tests were two tailed, and statistical significance was set as *P* < 0.05. SAS/ACCESS^®^ v. 9.4 (SAS Institute Inc., Cary, NC) was used for the analysis.

## RESULTS

Eight hospitals across seven countries (China, Japan, Singapore, Malaysia [two hospitals], Indonesia, India, and Pakistan) contributed data to this study. A total of 849 children suspected of having COVID-19 infection were enrolled between January 28, 2020 and November 6, 2020, of which 260 (30.6%) were laboratory-confirmed COVID-19 cases. Approximately half the cases were from Singapore (130 of 260, 50%). Laboratory confirmation was obtained via RT-PCR in 249 of 260 (95.8%) cases, IgM in 2 of 260 (0.8%) cases, and IgG seroconversion in 48 of 260 (18.5%) cases. The median (interquartile range) age of COVID-19 cases in the database was 6.5 (2.0, 12.0) years (Table 1). The median (interquartile range) age of COVID-19 cases was lowest in India [3.0 (1.0, 8.0) years] and highest in Indonesia [12.5 (11.0, 14.5) years]. There were slightly more male cases (about 50–60%) in most participating countries, except for Indonesia and Japan (25% and 33.%, respectively). In all countries, the most commonly reported contact history with COVID-19 cases was in household settings.

**Table 1:** Country-specific demographic characteristics of coronavirus disease 2019 cases and severity outcome in Asia

Demographics	Country							Severity		*P* value^*^
	China (*n* = 30)	Indonesia (*n* = 8)	India (*n* = 33)	Japan (*n* = 21)	Malaysia (*n* = 19)	Pakistan (*n* = 19)	Singapore (*n* = 130)	Mild/moderate (*n* = 230)	Severe/critical (*n* = 26)	
Age, y	10 (5–13)	12.5 (11–14.5)	3 (1–8)	4 (2–9)	6 (2–14)	4 (1–11)	7 (3–12)	7 (3–12)	4 (1–12)	0.204
Weight, kg	26.1 (10.0–26.1)	34.0 (24.0–39.5)	11.0 (7.5–24.0)	15.0 (12.0–17.0)	14.6 (10.6–25.0)	15.0 (9.0–35.0)	23.3 (12.9–44.8)	20.5 (12.0–36.5)	12.5 (6.5–36.0)	0.039
Male	17 (56.7)	2 (25.0)	17 (51.5)	7 (33.3)	10 (52.6)	17 (89.5)	73 (56.2)	123 (53.5)	18 (69.2)	0.148
Infants	2 (6.7)	0 (0.0)	10 (30.3)	2 (9.5)	2 (10.5)	4 (21.1)	8 (6.2)	18 (7.8)	8 (30.8)	0.002
Breastfeeding	0 (0.0)	0 (0.0)	7 (77.8)	1 (100)	2 (100)	2 (100)	3 (37.5)	10 (62.5)	5 (83.3)	0.616
Travel history	11 (36.7)	0 (0.0)	0 (0.0)	0 (0.0)	2 (10.5)	1 (5.3)	77 (59.2)	88 (38.6)	2 (14.3)	0.088
Exposure to confirmed case	27 (90.0)	5 (62.5)	24 (72.7)	20 (95.2)	16 (84.2)	0 (0.0)	115 (88.5)	200 (90.5)	5 (45.5)	< 0.001
Health care	1 (3.3)	1 (12.5)	0 (0.0)	0 (0.0)	0 (0.0)	0 (0.0)	4 (3.1)	5 (2.2)	1 (3.8)	0.478
School	0 (0.0)	0 (0.0)	0 (0.0)	0 (0.0)	1 (5.3)	0 (0.0)	1 (0.8)	2 (0.9)	0 (0.0)	1.000
Household	23 (76.7)	4 (50.0)	24 (72.7)	12 (57.1)	13 (68.4)	0 (0.0)	113 (86.9)	183 (79.6)	4 (15.4)	< 0.001
Other	2 (6.7)	0 (0.0)	0 (0.0)	8 (38.1)	2 (10.5)	0 (0.0)	2 (1.5)	14 (6.1)	0 (0.0)	0.373
Comorbidities	1 (3.3)	3 (37.5)	7 (21.2)	2 (9.5)	4 (21.1)	17 (89.5)	17 (13.1)	29 (12.6)	20 (76.9)	< 0.001
Cardiovascular	0 (0.0)	0 (0.0)	2 (6.1)	0 (0.0)	2 (10.5)	4 (21.1)	0 (0.0)	3 (1.3)	4 (15.4)	
Gastrointestinal	0 (0.0)	0 (0.0)	0 (0.0)	0 (0.0)	0 (0.0)	3 (15.8)	0 (0.0)	1 (0.4)	2 (7.7)	
Hematology/oncology	0 (0.0)	1 (12.5)	0 (0.0)	0 (0.0)	1 (5.3)	4 (21.1)	0 (0.0)	1 (0.4)	5 (19.2)	
Neurological	0 (0.0)	0 (0.0)	3 (9.1)	2 (9.5)	0 (0.0)	2 (10.5)	2 (1.5)	5 (2.2)	4 (15.4)	
Renal	0 (0.0)	0 (0.0)	1 (3.0)	0 (0.0)	0 (0.0)	1 (5.3)	0 (0.0)	1 (0.4)	1 (3.8)	
Respiratory	0 (0.0)	2 (25.0)	1 (3.0)	0 (0.0)	1 (5.3)	1 (5.3)	1 (0.8)	2 (0.9)	3 (11.5)	
Other	1 (3.3)	0 (0.0)	0 (0.0)	0 (0.0)	0 (0.0)	2 (10.5)	14 (10.8)	16 (7.0)	1 (3.8)	

Categorical variables are presented as count (percentage) and continuous variables are presented as median (interquartile range).

* The* P* value shows the differences between the two severity groups.

Records of clinical symptoms were available for 256 of 260 (98.5%) patients. At presentation, a significant proportion (102 of 256, 39.8%) of COVID-19 cases was asymptomatic (Table 2). The majority of these asymptomatic cases was identified through contact tracing and testing, as well as screening practices (e.g., screening for pre-operative patients**)**. Fever was the most common (98 of 154, (63.6%) symptom, followed by cough (60 of 154, 39.0%) and coryza (36 of 154, 23.4%). Lower respiratory signs at presentation were present in only 2 of 256 (< 1%) COVID-19 cases. Severe/critical cases were more likely to have fever, cough, poor feeding, diarrhea, vomiting, and seizures at presentation, compared with mild/moderate cases (Table 2). Laboratory investigations for patients were not routine in most centers’ patients (Table 3). Among cases with available laboratory investigations data, a lower hemoglobin level (9.5 g/dL [8.6, 11.4 g/dL] versus 13 g/dL [12.4, 13.8 g/dL]; *P* < 0.001), neutrophil count (2.9 neutrophils × 10^9^/L [2.1, 4.2 neutrophils × 10^9^/L] versus 5.2 neutrophils × 10^9^/L [3.5, 8.3 neutrophils × 10^9^/L]; *P* < 0.001), and albumin level (3.4 g/dL [2.4, 4.1 g/dL] versus 4.1 g/dL [3.9, 4.4 g/dL]; *P* = 0.002); and a higher alanine aminotransferase level [35.9 (20.0, 56.0) U/L versus 16.5 (13.0, 22.0) U/L; *P* = 0.002] and C-reactive protein level [116.0 (0.5, 179.1) mg/L versus 1.6 (0.2, 5.0) mg/L; *P* = 0.034] were associated with severe/critical disease compared with mild/moderate disease. Viral and bacterial co-infection were identified in only nine and seven cases, respectively.

**Table 2:** Clinical presentation of coronavirus disease 2019 cases and severity outcome in Asia

Clinical symptoms	Country							Severity		*P* value^*^
	China (*n* = 30)	Indonesia (*n* = 8)	India (*n* = 33)	Japan (*n* = 21)	Malaysia (*n* = 19)	Pakistan (*n* = 19)	Singapore (*n* = 130)	Mild/moderate (*n* = 230)	Severe/critical (*n* = 26)	
Asymptomatic	7 (30.4)	1 (25.0)	16 (57.1)	6 (37.5)	7 (36.8)	0 (0.0)	67 (58.8)	102 (52.6)	0 (0.0)	0.002
Fever	10 (33.3)	6 (75.0)	11 (33.3)	13 (61.9)	10 (52.6)	13 (68.4)	36 (27.7)	78 (33.9)	20 (76.9)	< 0.001
Cough	15 (50.0)	7 (87.5)	6 (18.2)	3 (14.3)	4 (21.1)	5 (26.3)	20 (15.4)	49 (21.3)	11 (42.3)	0.017
Coryza	4 (13.3)	1 (12.5)	1 (3.0)	3 (14.3)	2 (10.5)	0 (0.0)	26 (20.0)	36 (15.7)	1 (3.8)	0.105
Sore throat	1 (3.3)	1 (12.5)	0 (0.0)	0 (0.0)	0 (0.0)	0 (0.0)	13 (10.0)	15 (6.5)	0 (0.0)	0.180
Wheezing	0 (0.0)	0 (0.0)	0 (0.0)	0 (0.0)	0 (0.0)	0 (0.0)	0 (0.0)	0 (0.0)	0 (0.0)	NA
Crepitations	0 (0.0)	0 (0.0)	0 (0.0)	0 (0.0)	0 (0.0)	2 (10.5)	0 (0.0)	0 (0.0)	1 (3.8)	0.367
Headache	1 (3.3)	1 (12.5)	1 (3.0)	0 (0.0)	0 (0.0)	0 (0.0)	4 (3.1)	7 (3.0)	0 (0.0)	0.633
Irritability	0 (0.0)	0 (0.0)	0 (0.0)	0 (0.0)	0 (0.0)	0 (0.0)	2 (1.5)	2 (0.9)	0 (0.0)	0.008
Refuse feeding	0 (0.0)	2 (25.0)	0 (0.0)	0 (0.0)	1 (5.3)	1 (5.3)	0 (0.0)	2 (0.9)	2 (7.7)	0.001
Diarrhea	1 (3.3)	1 (12.5)	3 (9.1)	2 (9.5)	2 (10.5)	5 (26.3)	6 (4.6)	13 (5.7)	6 (23.1)	< 0.001
Vomiting	0 (0.0)	0 (0.0)	3 (9.1)	3 (14.3)	0 (0.0)	5 (26.3)	3 (2.3)	5 (2.2)	9 (34.6)	0.001
Seizures	0 (0.0)	0 (0.0)	1 (3.0)	0 (0.0)	0 (0.0)	2 (10.5)	0 (0.0)	1 (0.4)	2 (7.7)	< 0.001

NA = not applicable. Categorical variables are presented as count (percentage).

* The* P* value shows the differences between the two severity groups.

**Table 3:** Laboratory parameters of coronavirus disease 2019 cases and severity outcome in Asia

Laboratory parameters	Country							Severity		*P* value^*^
	China (*n* = 30)	Indonesia (*n* = 8)	India (*n* = 33)	Japan (*n *= 21)	Malaysia (*n* = 19)	Pakistan (*n* = 19)	Singapore (*n* = 130)	Mild/moderate (*n* = 230)	Severe/critical (*n* = 26)	
Hemoglobin, g/dL	13.1 (11.5–14.2)	13.0 (10.1–13.6)	10.8 (10.2–11.9)	12.8 (12.5–12.9)	12.8 (11.3–15.5)	9.3 (8.2, 11.4)	13.1 (12.4, 13.9)	13 (12.4, 13.8)	9.5 (8.6, 11.4)	< 0.001
WBC, ×10^9^ cells/L	6.2 (5.0– 8.2)	8.4 (4.9–14.6)	11.8 (5.4–23.3)	5.9 (5–10.8)	9.5 (8.6, 11.5)	10.3 (6.3, 15.6)	7.8 (6.4, 9.8)	7.6 (6.0, 10.0)	9.8 (6.3, 18.9)	0.053
Lymphocyte, ×10^9^/L	2.7 (2.0– 3.5)	3.0 (2.5–3.9)	4.9 (4.8–5.1)	2.9 (2.5–7.6)	4.0 (2.7–5.2)	2.7 (1.3–5.7)	3.3 (2.5–5.525)	3.2 (2.5–4.7)	4.3 (1.6–5.7)	0.744
Neutrophil, ×10^9^/L	2.1 (2.0– 2.6)	4.8 (3.8–9.9)	15.0 (1.6–15.2)	2.2 (1.7–2.2)	4.3 (1.6–6.8)	5.7 (4.6–10.6)	3.0 (2.2–4.1)	2.9 (2.1–4.2)	5.2 (3.5–8.3)	< 0.001
Platelets, ×10^9^/L	257 (227– 298)	336 (290–412)	139 (117–347)	254 (198–300)	278 (196–345)	233 (103–338)	334 (282–380)	318 (247–372)	295 (103–394)	0.249
APTT, s	26 (22.7–34.3)	34.7 (32.4–37.4)	32 (29.5–39.6)	35 (33.8–36.7)	29.6 (29.6–29.6)	28.8 (26.6–36.7)	41.8 (41.8–41.8)	33.5 (28.8–36.7)	29.7 (26.9–40.4)	0.700
PT, s	11.1 (10.8–11.6)	14 (13–14.5)	15 (14.4–15.5)	Not reported	10.6 (10.6–10.6)	13.2 (12.6–14.7)	13.5 (13.5–13.5)	13.3 (11.6–14.4)	13 (12.6–17.6)	0.457
INR	0.96 (0.93–1)	1 (0.915–1.11)	1.04 (0.97–1.2)	1.02 (1.02–1.04)	0.98 (0.98–0.98)	1.3 (1.2–1.4)	1.09 (1.09–1.09)	1.0 (1.0–1.1)	1.2 (1.2–1.7)	0.004
Total protein, g/dL	8.6 (6–8.7)	Not reported	6.1 (4.4–6.5)	6.9 (6.8–6.9)	7.6 (7.6–7.6)	Not reported	7.4 (7–7.8)	7.4 (7.0–7.8)	6.9 (5.7–7.2)	0.168
Albumin, g/dL	4.8 (4.5–5.2)	Not reported	3.8 (2.6–4.4)	4.4 (4.4–4.6)	3.9 (3.9–4.2)	3.2 (2.4–3.4)	4.1 (3.9–4.4)	4.1 (3.9–4.4)	3.4 (2.4–4.1)	0.002
Bilirubin, μmol/L	10.8 (5.3–13.7)	Not reported	0.6 (0.3–0.8)	0.4 (0.4–0.5)	4.7 (3.7–6.5)	6.8 (1.91–11.1)	6 (5–8)	6 (4–8)	9.1 (3.4–12.0)	0.406
AST, U/L	47 (22–48)	24 (19–28)	26.35 (21–35)	43 (39–43)	38 (22–59)	37.5 (28–122)	27 (21–35)	27 (21–37)	32 (21–39)	0.665
ALT, U/L	38 (33–55)	31 (25–44)	17.2 (9–50)	19 (17–20)	25 (16.5–41)	41 (23.5–61)	16 (13–21)	16.5 (13–22)	35.9 (20–56)	0.002
Urea, mmol/L	5.1 (1.3–8.3)	1.15 (1.1–1.2)	1.4 (0.8–1.6)	1.6 (0.8–5.1)	5.3 (5.1–6.6)	0.0 (3.6–8.9)	3.45 (2.7–4.1)	3.4 (1.4–5.1)	4.0 (1.1–8.9)	0.708
Sodium, mmol/L	137 (135–138)	145 (141–145)	141 (139–143)	138 (137–138)	140 (138–140)	136 (132–140)	141 (140–142)	139 (137–142)	138.7 (133–143)	0.950
Potassium, mmol/L	5.72 (5.6–5.9)	4.6 (4.4–5.1)	4.0 (3.2–4.5)	4.3 (4–4.4)	3.7 (3.7–3.9)	3.6 (3.3–3.8)	4.3 (4–4.7)	4.3 (3.8–4.6)	3.7 (3.3–3.9)	0.006
Creatinine, μmol/L	39.5 (23.4–50.1)	48 (48–49.5)	33 (22–40.5)	21 (21–25)	42.9 (35–44.1)	44.1 (35.4–106)	28 (20–47)	35 (26.52 to 48)	37.4 (26–106)	0.463
CRP, mg/L	1.0 (0.1–4.5)	5.0 (5.0–5.0)	0.4 (0.2–20.9)	3.2 (1.8–4.1)	0.2 (0.2–0.3)	179.1 (163.3–192.0)	1.2 (1.0–2.3)	1.6 (0.2–5.0)	116.0 (0.5–179.1)	0.034

ALT = alanine transaminase; APTT = activated thromboplastin time; AST = aspartate transaminase; CRP = C-reactive protein; INR = international normalized ratio; PT = prothrombin time; WBC = white blood cell count. Continuous variables are presented as median (interquartile range).

* The* P* value shows the differences between the two severity groups.

Outcome reporting was complete in 256 of 260 (98.5%) patients. Severe/critical COVID-19 infection was reported in 26 of 256 (10.2%) patients (Table 4). Infants (< 12 months) were more likely to develop severe/critical COVID-19 compared with older children (8 of 26 [30.8%] versus 18 of 230 [7.8%]; *P* = 0.002). The presence of a comorbidity was also associated with severe/critical disease compared with no comorbidity (20 of 26 [76.9%] versus 29 of 230 [12.6%]; *P* < 0.001).

**Table 4:** Outcomes of coronavirus disease 2019 cases in Asia

Outcomes	Country							Severity		*P* value^*^
	China (*n* = 30)	Indonesia (*n* = 8)	India (*n* = 33)	Japan (*n* = 21)	Malaysia (*n* = 19)	Pakistan (*n* = 19)	Singapore (*n* = 130)	Mild/moderate (*n* = 230)	Severe/critical (*n* = 26)	
Severity										
Mild	29 (96.7)	5 (62.5)	24 (77.4)	20 (95.2)	18 (94.7)	4 (22.2)	128 (99.2)			
Moderate	0 (0.0)	0 (0.0)	2 (6.5)	0 (0.0)	0 (0.0)	0 (0.0)	0 (0.0)			
Severe	1 (3.3)	3 (37.5)	3 (9.7)	1 (4.8)	1 (5.3)	12 (66.7)	1 (0.8)			
Critical	0 (0.0)	0 (0.0)	2 (6.5)	0 (0.0)	0 (0.0)	2 (11.1)	0 (0.0)			
Oxygen therapy	1 (20.0)	3 (37.5)	3 (9.4)	1 (4.8)	0 (0.0)	10 (55.6)	1 (0.8)	0 (0.0)	13 (92.9)	< 0.001
HFNC	0 (0.0)	0 (0.0)	0 (0.0)	0 (0.0)	1 (5.9)	3 (16.7)	0 (0.0)	0 (0.0)	2 (12.5)	0.005
CPAP	0 (0.0)	0 (0.0)	1 (3.1)	1 (4.8)	0 (0.0)	1 (5.6)	0 (0.0)	0 (0.0)	1 (5.9)	0.077
BiPAP	0 (0.0)	0 (0.0)	0 (0.0)	0 (0.0)	0 (0.0)	2 (11.1)	0 (0.0)	0 (0.0)	02 (10.5)	0.007
Mechanical ventilation	0 (0.0)	0 (0.0)	3 (9.4)	0 (0.0)	0 (0.0)	3 (16.7)	0 (0.0)	0 (0.0)	6 (24.0)	< 0.001
Organ dysfunction										
Cardiovascular	1 (3.3)	0 (0.0)	2 (6.3)	0 (0.0)	0 (0.0)	2 (11.1)	0 (0.0)	0 (0.0)	5 (20.0)	< 0.001
Pulmonary	0 (0.0)	2 (25.0)	1 (3.1)	0 (0.0)	0 (0.0)	9 (50.0)	0 (0.0)	0 (0.0)	12 (48.0)	< 0.001
Neurological	0 (0.0)	0 (0.0)	3 (9.4)	0 (0.0)	0 (0.0)	2 (11.1)	0 (0.0)	2 (0.9)	3 (12.0)	0.007
Hepatic	0 (0.0)	0 (0.0)	0 (0.0)	0 (0.0)	0 (0.0)	1 (5.6)	2 (1.6)	2 (0.9)	1 (4.0)	0.267
Renal	0 (0.0)	0 (0.0)	1 (3.1)	0 (0.0)	0 (0.0)	4 (22.2)	0 (0.0)	1 (0.4)	4 (16.0)	0.000
Hematological	0 (0.0)	0 (0.0)	0 (0.0)	0 (0.0)	1 (5.3)	5 (27.8)	0 (0.0)	2 (0.9)	4 (16.0)	0.001
Hospital duration, d	13 (9, 17)	8 (7, 17)	15 (11, 19)	6.5 (5, 9)	9 (8, 12)	7 (4, 13)	11 (8, 20)	11 (8, 19)	8 (6, 12)	0.014
Highest inpatient status										
General ward	3 (60.0)	7 (87.5)	26 (78.8)	10 (90.9)	17 (100)	0 (0.0)	130 (100)	188 (97.4)	4 (16.0)	< 0.001
Intermediate care	1 (20.0)	0 (0.0)	3 (9.1)	1 (9.1)	0 (0.0)	0 (0.0)	0 (0.0)	1 (0.5)	3 (12.0)	
ICU	1 (20.0)	1 (12.5)	4 (12.1)	0 (0.0)	0 (0.0)	18 (100)	0 (0.0)	4 (2.1)	18 (72.0)	
Mortality	0 (0.0)	0 (0.0)	1 (3.1)	0 (0.0)	0 (0.0)	5 (27.8)	0 (0.0)	0 (0.0)	6 (25.0)	<0.001

BiPAP = bilevel positive airway pressure; CPAP = continuous positive airway pressure; HFNC = high-flow nasal cannula; ICU = intensive care unit. Categorical variables are presented as count (percentage) and continuous variables are presented as median (interquartile range).

* The* P* value shows the differences between the two severity groups.

Pulmonary organ dysfunction occurred in 12 patients, six of whom required invasive respiratory support, and five fulfilled criteria for pediatric acute respiratory distress syndrome (Table 4). Non-invasive respiratory support, including oxygen, was required in five patients. Organ dysfunctions were also seen in other systems (Table 4). The overall mortality was 6 of 256 (2.3%) cases, all of which occurred in Pakistan (*n* = 5) and India (*n* = 1). There were no cases of MIS-C in this cohort. In the multivariable logistic regression, infant age group (adjusted odds ratio [aOR], 4.65; 95% CI, 1.90–11.38), presence of comorbidities (aOR, 8.08; 95% CI, 1.79–36.41), and cough on presentation (aOR, 2.41; 95% CI, 1.32–4.39)] were associated with severe/critical COVID-19 infection (Table 5).

**Table 5:** Multivariable analysis of predictive factors for severe/critical coronavirus disease 2019 in Asian children

Variables	Univariate			Multivariate		
	OR	95% CI	*P* value	OR	95% CI	*P* value
Infant	2.38	1.26–4.88	0.008	4.65	1.90–11.38	< 0.001
Comorbidities	3.82	1.03–14.18	0.045	8.08	1.79–36.41	0.007
Cough	1.60	1.14–2.24	0.006	2.41	1.32–4.39	0.004

OR = odds ratio; CI = confidence interval.

## DISCUSSION

This epidemiological study of pediatric COVID-19 infections found that the common presenting clinical signs and symptoms of COVID-19 in children in our Asian hospital network were similar: fever, cough, and coryza. The overall asymptomatic rate was about 40%. With the exception of Pakistan, which only included cases admitted to the ICU, children with COVID-19 also had similar minimal abnormalities in laboratory parameters at presentation. Pediatric exposure to SARS-CoV-2 was reported to occur mainly in household settings. The rate of severe/critical COVID-19 disease was 0.8% to 5.3% in Singapore, Malaysia, Japan, and China compared with 16% in India, 37.5% in Indonesia, and 78% in Pakistan. The higher rates in India, Indonesia, and Pakistan were possibly a manifestation of testing and hospitalization prioritization for children with more severe clinical manifestation. No children with confirmed COVID-19 met the MIS-C criteria in our registry. Overall mortality was 2.3% (6 of 256) in our registry, with all deaths reported from India and Pakistan. Infant age group (< 12 months), the presence of comorbidities, and cough on presentation were identified as risk factors associated with severe/critical COVID-19 infection in children.

In our regional registry, pediatric COVID-19 symptoms on presentation were mostly consistent with an upper respiratory tract infection, with fever, cough, and coryza being the most common symptoms. Transmission was mostly reported to occur in household settings. As some countries may have enforced nation-wide lock downs or school closures, the risk of SARS-CoV-2 transmission in the school setting was not apparent during this study period. However, published studies have reported the low risk of SARS-CoV-2 transmission in educational settings as well as differences in infection rates within households by age group.^17–19^ The prevalence of gastrointestinal symptoms including diarrhea, vomiting, and poor feeding, which have been associated with MIS-C, were also associated with severe/critical infection.^20^ Raised C-reactive protein values in COVID-19 cases were only evident in Pakistan, which is not surprising because all cases were admitted to the ICU. Evidence of significant systemic inflammatory response during the infection is known to be associated with severe disease in adults. None of the cases in our registry fulfilled the criteria for MIS-C. However, our study was not designed specifically to investigate MIS-C, which can occur weeks after SARS-CoV-2 infection, and RT-PCR/IgM may no longer be positive.^5^

The epidemiologic profiles of cases among each represented country were different. In Singapore, we observe that the majority of cases were associated with travel (59%) and being exposed to a positive household close contact (87%). In Malaysia, however, the nationwide movement restriction order likely accounted for the lower proportion of travel-associated cases (10.5%).^21–23^ In contrast, in Pakistan, despite publishing mitigation guidelines (*National Action Plan for Corona Virus Disease [COVID-19] Pakistan*^24^), transmission in the form of local community spread of mostly asymptomatic cases was observed.^25^ The presence of comorbidities was also greater in countries such as Pakistan (90%) and Indonesia (38%), compared with China (3%), Japan (10%), Singapore (13%), and Malaysia (21%). It is not entirely clear from this registry study why these centers had a greater proportion of patients with comorbidities, although we suspect it could be a result of admission prioritization for children with a more severe clinical manifestation in the group with comorbidities, or an exacerbation or increased need for medical care resulting from the underlying comorbid condition itself during the pandemic.

Health-care systems and public health pandemic response to COVID-19 in Asia are highly variable. In Singapore, contact tracing and diagnostic testing are widespread, and all confirmed COVID-19 cases were isolated institutionally until they were deemed non-infective before patients were discharged from the hospital, leading to a prolonged duration of hospital stay median (interquartile range) of 11 (8, 20) days, despite the majority of cases being asymptomatic (67 of 130, 59%).^26,27^ Similarly, many of the patients in Japan and Malaysia were asymptomatic, and only one case each required continuous positive airway pressure or high-flow nasal cannula, respectively. In Malaysia, patients were kept in the hospital until 14 days after the onset of symptoms. In Japan and Malaysia, the median (interquartile range) hospital stay was 6.5 (5.0, 9.0) and 9 (8.0, 12.0) days, respectively. In contrast, the recruiting center in Pakistan, which was situated in Karachi, the largest urban city of Pakistan, had the highest case density in the country. This unit received referrals of patients with underlying acute and chronic diseases from other hospitals, many of whom were found to test positive for COVID-19 infection. Cases referred here were unlike the asymptomatic cases identified from contact tracing (these were advised to quarantine at home). On the contrary, patients seen here had significant symptoms warranting admission to the pediatric ICU. A high proportion of these patients developed organ dysfunction (82%) and required respiratory support (78%). Mortality in this unit was 26%.

Although differences in testing capacity, health systems, and pandemic response strategies across Asia are potential reasons for the differences described, differences in COVID-19 infection rates and outcomes could also be present as a result of genetic factors, cultural practices, environmental exposures, or combinations of biological and social factors. For example, angiotensin converting enzyme-2 receptor polymorphisms exist across gender and races, and have been implicated in affecting the severity of COVID infection in different regions.^28^ The results of our study indicate that, even within the “Asian” racial group, these differences may exist, and in-depth studies to disentangle the role of each contributing factor is vital. India^8^ and Pakistan^9^ have previously reported cases of MIS-C, whereas none of the severe/critical cases in our registry from Singapore, Malaysia, Indonesia, Japan, and China met the criteria for MIS-C. Our study also suggests that extrapolation of data from other geographic or racial populations may be inappropriate, and further investigations should include Asian representation.

This study leverages on a multi-center research network to include data from seven countries across Asia. All included sites are national COVID-19 referral centers. Nevertheless, the number of sites included were few and may not be fully representative of all cases in each country. Individual centers/countries have unique health-care policies in managing this pandemic, and criteria for screening suspected cases, indications for hospital admission, duration of stay, need for laboratory testing, and so on may differ and bias our results. Representation from northern regions of southeast Asia including Thailand, Laos, Cambodia, and Myanmar, which have unique cultures, demographics, and health-care profiles, are missing. Sites were encouraged to enter consecutive patient’s data on a weekly basis to ensure up-to-date reporting; however, as this was not monitored and mandated, it may have resulted in non-consecutive enrollments or missing data. The inclusion of only ICU-level patients from Pakistan may have biased the results. Bias may have also been introduced because this study used passive surveillance methods that were reliant on reporting by the respective hospitals. The possibility of false-positive/false-negative COVID-19 PCR, IgM, or IgG results cannot be excluded, although the majority of our cases were confirmed via PCR, which is known to have high sensitivity and specificity. Last, after discharge from the hospital, this study did not include any further follow-up visits. Therefore, intermediate to long-term complications were not captured.

## CONCLUSION

The clinical presentation and basic laboratory parameters of SARS-CoV-2 infection in children from seven Asian countries were similar. The overall asymptomatic rate was about 40%, and mortality was 2.3%, with all deaths reported from Pakistan and India. No cases of MIS-C were identified in our study. Risk factors for severe/critical pediatric COVID-19 infection were found to be age < 12 months, cough at presentation, and presence of comorbidities. Further data will be accrued in the PACCOVRA registry to determine the impact of pediatric COVID-19 over time and over larger geographic regions in Asia.

## References

[1] Dong Y 2020. Epidemiology of COVID-19 among children in China. Pediatrics 2020: e20200702. 10.1542/peds.2020-0702. 32179660

[2] Shekerdemian LS, 2020. Characteristics and outcomes of children with coronavirus disease 2019 (COVID-19) infection admitted to US and Canadian pediatric intensive care units. JAMA Pediatr 174: 868–873. 10.1001/jamapediatrics.2020.1948 PMC7489842 32392288

[3] Bellino S 2020. COVID-19 disease severity risk factors for pediatric patients in Italy. Pediatrics 146: e2020009399. 10.1542/peds.2020-009399. 32665373

[4] Hoang A 2020. COVID-19 in 7780 pediatric patients: a systematic review. EClinicalMedicine 24: 100433. 10.1016/j.eclinm.2020.100433. 32766542 PMC7318942

[5] Whittaker E 2020. Clinical characteristics of 58 children with a pediatric inflammatory multisystem syndrome temporally associated with SARS-CoV-2. JAMA 324: 259–269. 32511692 10.1001/jama.2020.10369 PMC7281356

[6] Verdoni L 2020. An outbreak of severe Kawasaki-like disease at the Italian epicentre of the SARS-CoV-2 epidemic: an observational cohort study. Lancet 395: 1771–1778. 32410760 10.1016/S0140-6736(20)31103-X PMC7220177

[7] Feldstein LR 2020. Multisystem inflammatory syndrome in U.S. children and adolescents. N Engl J Med 383: 334–346. 32598831 10.1056/NEJMoa2021680 PMC7346765

[8] Jain S 2020. Multisystem inflammatory syndrome in children with COVID-19 in Mumbai, India. Indian Pediatr 57: 1015–1019. 32788432 10.1007/s13312-020-2026-0 PMC7678602

[9] Sadiq M 2020. Multisystem inflammatory syndrome associated with COVID-19 in children in Pakistan. Lancet Child Adolesc Health 4: e36–e37. 32791052 10.1016/S2352-4642(20)30256-X PMC7417160

[10] Wong JJM, 2021. Changes adopted in Asian pediatric hospitals during the COVID-19 pandemic: a report from the Pediatric Acute and Critical Care COVID-19 Registry of Asia. J Pediatr Intensive Care (Epub ahead of print). 10.1055/s-0040-1722340. PMC9381435 35990879

[11] von Elm E Altman DG Egger M Pocock SJ Gøtzsche PC Vandenbroucke JP , 2007. The Strengthening the Reporting of Observational Studies in Epidemiology (STROBE) statement: guidelines for reporting observational studies. Prev Med 45: 247–251. 17950122 10.1016/j.ypmed.2007.08.012

[12] Li J 2020. Comparative analysis of symptomatic and asymptomatic SARS-CoV-2 infection in children. Ann Acad Med Singap 49: 530–537. 33164022

[13] Nallasamy K 2021. Clinical profile, hospital course and outcome of children with COVID-19. Indian J Pediatr 2021: 1–6. 10.1007/s12098-020-03572-w. PMC7881747 33580873

[14] Harris PA Taylor R Thielke R Payne J Gonzalez N Conde JG , 2009. Research electronic data capture (REDCap): a metadata-driven methodology and workflow process for providing translational research informatics support. J Biomed Inform 42: 377–381. 18929686 10.1016/j.jbi.2008.08.010 PMC2700030

[15] World Health Organization , 2020. Clinical Management of COVID-19: Interim Guidance. Geneva, Switzerland: WHO.

[16] Goldstein B Giroir B Randolph A , 2005. International pediatric sepsis consensus conference: definitions for sepsis and organ dysfunction in pediatrics. Pediatr Crit Care Med 6: 2–8. 15636651 10.1097/01.PCC.0000149131.72248.E6

[17] Yung CF 2020. Household transmission of severe acute respiratory syndrome coronavirus 2 from adults to children. J Pediatr 225: 249–251. 32634405 10.1016/j.jpeds.2020.07.009 PMC7334921

[18] Yung CF 2021. Novel coronavirus 2019 transmission risk in educational settings. Clin Infect Dis 72: 1055–1058. 32584975 10.1093/cid/ciaa794 PMC7337629

[19] Ismail SA Saliba V Lopez Bernal J Ramsay ME Ladhani SN , 2021. SARS-CoV-2 infection and transmission in educational settings: a prospective, cross-sectional analysis of infection clusters and outbreaks in England. Lancet Infect Dis 21: 344–353. 33306981 10.1016/S1473-3099(20)30882-3 PMC7833602

[20] Jiang L 2020. COVID-19 and multisystem inflammatory syndrome in children and adolescents. Lancet Infect Dis 20: e276–e288. 32818434 10.1016/S1473-3099(20)30651-4 PMC7431129

[21] Tang KHD , 2020. Movement control as an effective measure against COVID-19 spread in Malaysia: an overview. Z Gesundh Wiss 2020 Jun 13: 1–4. 10.1007/s10389-020-01316-w. PMC7293423 32837842

[22] Ng CFS Seposo XT Moi ML Tajudin M Madaniyazi L Sahani M , 2020. Characteristics of COVID-19 epidemic and control measures to curb transmission in Malaysia. Int J Infect Dis 101: 409–411. 33075527 10.1016/j.ijid.2020.10.027 PMC7567666

[23] Shah AUM 2020. COVID-19 outbreak in Malaysia: actions taken by the Malaysian government. Int J Infect Dis 97: 108–116. 32497808 10.1016/j.ijid.2020.05.093 PMC7264933

[24] Ministry of National Health Services, Regulation, and Coordination, Government of Pakistan, 2020. *National Action Plan for Corona Virus Disease (COVID-19) Pakistan*. Available at: https://www.nih.org.pk/wp-content/uploads/2020/03/COVID-19-NAP-V2-13-March-2020.pdf.

[25] Javed B Sarwer A Soto EB Mashwani ZuR , 2020. Is Pakistan’s response to coronavirus (SARS-CoV-2) adequate to prevent an outbreak? Front Med 7: 158. 10.3389/fmed.2020.00158. PMC7187779 32373620

[26] Chia ML Him Chau DH Lim KS Yang Liu CW Tan HK Tan YR , 2020. Managing COVID-19 in a novel, rapidly deployable community isolation quarantine facility. Ann Intern Med 174: 247–251. 32941059 10.7326/M20-4746 PMC7505018

[27] Chua AQ 2020. Health system resilience in managing the COVID-19 pandemic: lessons from Singapore. BMJ Glob Health 5: e003317. 10.1136/bmjgh-2020-003317 PMC7496566 32938609

[28] Khayat AS 2021. ACE2 polymorphisms as potential players in COVID-19 outcome. PLoS One 15: e0243887. 10.1371/journal.pone.0243887 PMC7769452 33370311

